# Mimicking Axon Growth and Pruning by Photocatalytic Growth and Chemical Dissolution of Gold on Titanium Dioxide Patterns

**DOI:** 10.3390/molecules30010099

**Published:** 2024-12-30

**Authors:** Fatemeh Abshari, Moritz Paulsen, Salih Veziroglu, Alexander Vahl, Martina Gerken

**Affiliations:** 1 Chair for Integrated Systems and Photonics, Department of Electrical and Information Engineering, Faculty of Engineering, Kiel University, Kaiserstr. 2, 24143 Kiel, Germany; 2 Chair for Multicomponent Materials, Department of Materials Science, Faculty of Engineering, Kiel University, Kaiserstr. 2, 24143 Kiel, Germany; 3 Kiel Nano, Surface and Interface Science (KiNSIS), Kiel University, Christian-Albrechts-Platz 4, 24118 Kiel, Germany; 4 Leibniz Institute for Plasma Science and Technology, Felix-Hausdorff-Str. 2, 17489 Greifswald, Germany

**Keywords:** photocatalytic deposition, chemical dissolution, gold, titanium dioxide, potassium iodide solution, indium tin oxide, neuromorphic engineering, transmission optical microscopy

## Abstract

Biological neural circuits are based on the interplay of excitatory and inhibitory events to achieve functionality. Axons form long-range information highways in neural circuits. Axon pruning, i.e., the removal of exuberant axonal connections, is essential in network remodeling. We propose the photocatalytic growth and chemical dissolution of gold lines as a building block for neuromorphic computing mimicking axon growth and pruning. We predefine photocatalytic growth areas on a surface by structuring titanium dioxide (TiO_2_) patterns. Placing the samples in a gold chloride (HAuCl_4_) precursor solution, we achieve the controlled growth of gold microstructures along the edges of the indium tin oxide (ITO)/TiO_2_ patterns under ultraviolet (UV) illumination. A potassium iodide (KI) solution is employed to dissolve the gold microstructures. We introduce a real-time monitoring setup based on an optical transmission microscope. We successfully observe both the growth and dissolution processes. Additionally, scanning electron microscopy (SEM) analysis confirms the morphological changes before and after dissolution, with dissolution rates closely aligned to the growth rates. These findings demonstrate the potential of this approach to emulate dynamic biological processes, paving the way for future applications in adaptive neuromorphic systems.

## 1. Introduction

Neuromorphic engineering focuses on developing advanced computational systems by drawing inspiration from the structure and processes of biological neural networks, with the goal of achieving improved efficiency [1]. In neural networks, neuronal connections are dynamically and continuously reorganized. These connections evolve over different time scales: rapid synaptic plasticity results in localized adjustments at synapses between neurons, while slower processes, such as axon growth and pruning, occur throughout the broader network. Building on the dynamic reorganization of neuronal connections, axon growth represents a critical phase of neural network development, where intrinsic genetic programs and extracellular signals work in concert to extend axons toward their target regions, forming the foundational pathways for neural circuitry [2,3]. Many studies have focused on replicating rapid synaptic plasticity using memristive devices, given their unique ability to enable in-memory computing [4,5]. Ongoing research continues to investigate neuronal connections at a global scale within biological neural networks, while simultaneously developing innovative methods to integrate these mechanisms into next-generation bio-inspired systems [6].

Recent advances in neuromorphic engineering have increasingly focused on nanowire networks that exhibit memristive properties, which are being explored for their ability to emulate the dynamic and adaptable behavior of synapses in biological neural networks. These networks are gaining attention for their potential to enhance computational efficiency by replicating both short-term synaptic plasticity and long-term memory storage, paving the way for the integration of such systems into bio-inspired computing architecture [7,8,9]. These networks are naturally self-organizing, with nanowires forming conductive, one-dimensional (1D) pathways. The intricate topology of these networks results in collective switching behaviors, making them highly compatible with memristive architecture. In biological neural networks, the ability to dynamically regulate stimuli and control the formation and dissolution of connections is a key feature for adaptive functionality [10,11,12].

Axonal pruning is a critical process in the development and remodeling of neural networks, wherein excess or improperly connected axons are selectively eliminated to refine the neural circuitry, ensuring the optimal function of the nervous system [13]. This process occurs primarily during developmental stages, but also plays a role in adult neuroplasticity, where axonal pruning helps to fine-tune neural pathways based on learning, experience, and environmental stimuli [13]. Mechanistically, axonal pruning is regulated by molecular signals that induce synaptic weakening and the targeted retraction of axons, a process that parallels cellular processes, like apoptosis, ultimately contributing to the structural and functional optimization of neural circuits [14]. In the early stages, attempts to replicate the global interactions within neuronal assemblies were made by exploring global connectivity through electrolyte gating in liquid media [15].

We focus on the gradual formation of one-dimensional, long-range connections, which have the potential to enable adaptive modifications in network topology. This aspect of the research aims to mimic axonal growth through the photocatalytic deposition of conductive gold lines onto ultraviolet (UV) light-activated titanium dioxide (TiO_2_) substrates. Additionally, we explore the pruning of these axonal-like structures by the chemical dissolution of the grown gold lines using a potassium iodide (KI) solution, simulating the axonal pruning process. Chemical dissolution was chosen as it allows for a gradual loss of conductivity in continuous gold lines due to the reduction in the gold line diameter. Once the gold coverage falls below the percolation threshold, conductivity is lost completely. With gold growth, the percolation threshold may be reached again, regaining conductivity. Therefore, this approach promises a reversible, stimulus-dependent growth and pruning of network connections during the learning process mimicking the situation in biological neural networks. Here, it is recognized that the density of the human dendrite network increases from birth to the age of 2 years old [16]. Subsequently, the density decreases again, which is associated with network consolidation during learning.

TiO_2_ is widely utilized as a semiconductor photocatalyst due to its high photocatalytic efficiency, ease of fabrication, cost-effectiveness, non-toxic nature, and robust chemical stability [17,18,19]. Considering the critical role of TiO_2_ as a semiconductor photocatalyst with excellent optical properties, recent studies have demonstrated its ability to facilitate photocatalytic processes under UV light, particularly for structural modifications and degradation mechanisms [20]. It was recently shown that the co-precipitation of TiO_2_ with terbium and manganese effectively improves its photocatalytic performance by lowering the band gap energy and enhancing electron-hole separation, which facilitates the efficient degradation of tetracycline antibiotics under both UV- and visible-light conditions [21]. The deposition of gold onto TiO_2_ thin films has been successfully achieved through the photoreduction of gold chloride (HAuCl_4_) under UV illumination [22,23,24]. It has been reported that the addition of isopropanol to the HAuCl_4_ solution can significantly accelerate the photoreduction process by acting as a hole scavenger, thereby enhancing the speed of gold nanoparticle growth under UV light [25]. In addition to the use of isopropanol, other parameters have been shown to significantly influence the morphology and coverage of the deposited gold structures. These include factors such as the crystal structure and surface morphology of the underlying TiO_2_ thin film, the composition and pH of the precursor solution, as well as the intensity and duration of UV illumination [26,27].

Through UV illumination, TiO_2_ facilitates the generation of electron-hole pairs, with electrons migrating to the conduction band. These electrons reduce Au^3+^ ions from the HAuCl_4_ solution, leading to the formation of neutral gold atoms The gold atoms form solid nuclei on the TiO_2_ surface, which then grow through the consecutive addition of gold atoms into gold nano- and microparticles. Simultaneously, the holes oxidize nearby molecules, completing the photocatalytic reaction [28]. The efficiency of photocatalytic processes, such as gold growth on TiO_2_ surfaces, is strongly influenced by light intensity, as higher UV intensities enhance the generation of electron-hole pairs, accelerating reaction rates and structural formation [29]. These findings provide valuable insights into how UV illumination affects both the growth and morphological evolution of gold particles [30].

**Figure 1:**
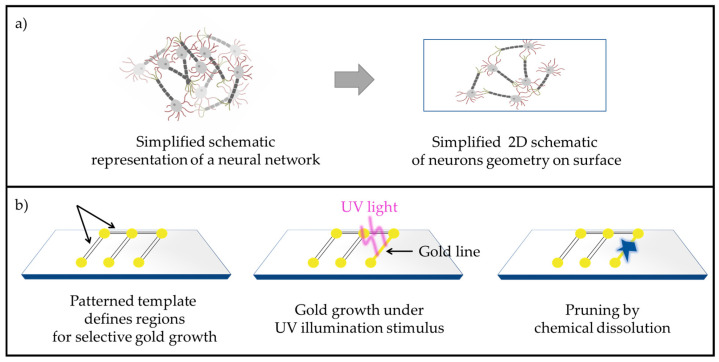
(**a**) Simplified schematic representation of a neural network (**left**) and its abstraction into 2D schematic geometry on a surface (**right**), retaining the essential connectivity of the network. (**b**) Process for mimicking axon-like connections: (**left**) a patterned template defines regions for selective gold growth, (**center**) UV illumination induces photocatalytic gold growth along predefined pathways, forming axon-like structures, and (**right**) chemical dissolution selectively prunes the gold connections, mimicking axonal pruning.

In this study, photocatalysis is utilized to grow gold lines on TiO_2_ thin films patterned using lithography. A thin indium tin oxide (ITO) sublayer is incorporated beneath the TiO_2_. Given that ITO has a higher work function compared to TiO_2_, a Schottky barrier forms at the TiO_2_-ITO interface, which affects the overall photocatalytic performance [31,32]. In a recent study, we demonstrated that a 6 nm ITO layer beneath TiO_2_ effectively promotes localized gold growth along the edges of the TiO_2_ patterns resulting in electrically conductive gold lines [33]. This study builds on these previous findings by using a consistent template with a 6 nm ITO layer beneath the TiO_2_ for all experiments. Here, we investigate the sequential processes of photocatalytic gold growth followed by chemical dissolution mimicking axon growth and pruning. The schematic in Figure 1 demonstrates the abstraction process for mimicking neuronal network dynamics through a material-based approach. Figure 1a on the left presents a simplified schematic representation of a neural network, illustrating the interconnectivity between neurons in a network. This is not a true 3D neuronal network, but rather a simplified projection to communicate the idea of neuronal connectivity. Moving to the right, the neural network is translated into a 2D schematic geometry on a surface. This 2D representation simplifies the complex volumetric arrangement while retaining the essential connectivity, making it accessible for technical implementation.

Figure 1b introduces the technical methodology used to mimic axon-like connections in a proposed material system. First, a patterned template is designed to define specific regions for selective gold growth. This template mimics synapse-like nodes connected by axon-like linear pathways. Under UV illumination, the photocatalytic growth of gold structures is induced along these defined pathways, simulating the formation of axon-like connections. The resulting gold pathways provide a physical basis for mimicking neuronal connectivity in a controllable manner. Finally, the grown gold structures undergo a targeted chemical dissolution process, emulating the natural phenomenon of axonal pruning. This selective removal of gold pathways enables dynamic modifications of the network, reflecting the adaptive and self-organizing properties of biological neural circuits. This approach represents a proposed material system to mimic the formation and dissolution of axon-like connections in a simplified, reproducible framework. By combining photocatalytic growth and chemical dissolution processes, this study introduces a methodology for investigating adaptive, neuromorphic systems, laying the groundwork for further exploration in network remodeling.

To further clarify this, the abstraction process depicted in Figure 1 bridges the gap between biological complexity and material implementation. The transition from a neural network to a patterned material system involves translating the functional principles of connectivity and adaptability into a simplified, physical framework. This includes using the patterned template to mimic axonal pathways, where gold deposition represents axonal growth and its selective removal represents pruning. By replicating these key features, the proposed system provides a versatile platform to study the dynamic remodeling of networks, offering insights into neuromorphic engineering and adaptive material systems.

The chemical dissolution of gold using the KI solution has proven to be an effective method for removing gold from various substrates. KI acts as a complexing agent, forming soluble gold–iodine complexes, which enable the controlled removal of gold lines grown during photocatalytic processes [34]. This dissolution process mimics the axonal pruning in neural networks, allowing for repeated cycles of growth and removal, essential for dynamic neuromorphic systems. In this paper, we aim to investigate the UV-stimulated photocatalytic growth of gold on titanium dioxide patterns, followed by chemical dissolution using KI solutions. By analyzing both real-time optical monitoring data and post-experiment scanning electron microscopy (SEM) imaging, we provide insights into the mechanisms governing these processes and their potential for precise microstructure manipulation.

## 2. Results

### 2.1. Analysis of Gold Growth Dynamics on the Edge and Surface of TiO_2_

**Figure 2:**
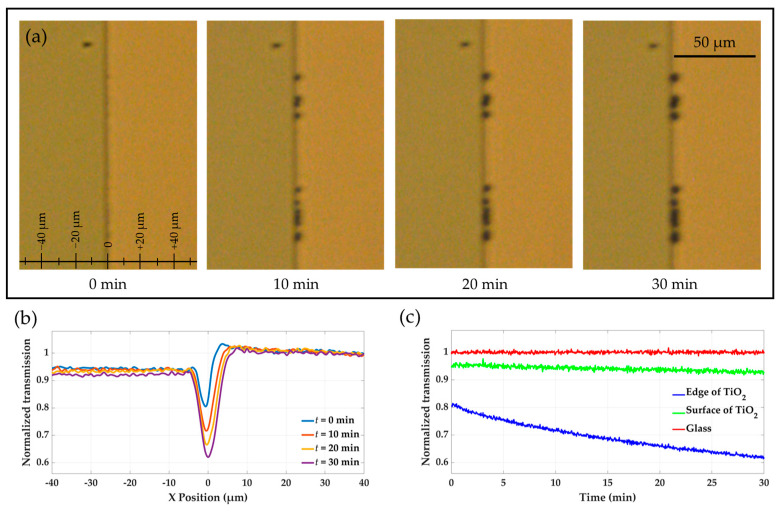
Real-time monitoring of gold growth (**a**) Sequential optical microscope images of a selected region around the TiO_2_ edge, including the TiO_2_ surface on the left side and the glass substrate on the right side, taken at various time points, *t* = 0, *t* = 10, *t* = 20, and *t* = 30 min, illustrating the gradual growth of gold structures over 30 min. (**b**) Transmission profiles across the TiO_2_ edge at *t* = 0, *t* = 10, *t* = 20, and *t* = 30 min, normalized to the reference transmission on the glass substrate. (**c**) Time-dependent transmission curves showing dissolution progress at the TiO_2_ edge and surface compared to the glass substrate.

In this section, the photocatalytic growth of gold lines on TiO_2_ edges is analyzed in real-time using optical transmission microscope data. As described in Section 4, a beaker was filled with 20 mL of a gold chloride precursor solution, mixed with isopropanol in a 10:1 ratio to enhance the deposition rate. The substrate was submerged in this solution and exposed to UV illumination (λ = 365 nm) for 30 min. The growth process was monitored through a transmission microscope by capturing images every 4 s over a total duration of 30 min. A certain region around the TiO_2_ edge was selected where the gold structures predominantly grew. The selected region included the TiO_2_ surface on the left side and the glass substrate on the right side of the TiO_2_ edge located in the center. Figure 2a presents optical microscope images of the selected region taken at different times during the growth process: *t* = 0, *t* = 10, *t* = 20, and *t* = 30 min. A supplementary video (Video S1) is provided to offer a clearer visualization of the growth dynamics.

For every pixel of the selected region, we measured a distinct transmitted light intensity. At each *x*-coordinate, we averaged the measured intensity values over all pixels with different *y*-coordinates to generate transmission profiles across the *x*-span of the selected region. These transmission curves, showing the variation in intensity as a function of *x*-span, are plotted in Figure 2b for four different time points: *t* = 0, *t* = 10, *t* = 20, and *t* = 30 min. All transmission values are normalized relative to the reference transmission on the bare glass substrate, located far from the TiO_2_ edge at *x* = 40 µm.

At the beginning of the growth process (*t* = 0), the transmission at the TiO_2_ edge (*x* = 0) was lower than those of the TiO_2_ surface (*x* < 0) and the glass substrate (*x* > 0). This difference is attributed to light scattering at the TiO_2_ edge, which creates a narrow dark line in the middle of the selected area, as displayed in the optical microscopy images (Figure 2a). Furthermore, the transmission on the TiO_2_ surface (*x* < 0) was slightly lower than that of the glass substrate (*x* > 0). During the growth process, a decrease in the transmitted light intensity is observed for *x* = 0 and *x* < 0, corresponding to the growth of gold particles along the edge and on the surface of TiO_2_ over time. At the end of the growth experiment (*t* = 30 min), the normalized transmission at the edge (*x* = 0) was significantly reduced compared to the beginning time (*t* = 0), with a total change of Δ*T*_Edge_ ≈ 0.19 over 30 min. On the surface of the TiO_2_ (*x* < 0), the transmission was slightly reduced, with Δ*T*_Surface_ ≈ 0.03 after 30 min of growth. While a significant growth rate is achieved along the edge of TiO_2_, little changes in transmission are observed on the left and right sides of the edge, confirming the selective formation of gold lines along the edge.

The dissolution process of gold structures on the TiO_2_ edge and surface was analyzed by monitoring transmission changes over time. The glass substrate served as a reference, as it remained unaffected throughout the experiment. The transmission at the TiO_2_ edge and surface increased during dissolution, indicating the gradual removal of gold structures. Figure 2 part (c) of the figure presents the time-dependent transmission curves for these regions, highlighting the sharper rise in transmission at the TiO_2_ edge compared to the surface, reflecting the higher density of gold structures at the edge.

### 2.2. Analysis of Gold Dissolution Dynamics on the Edge and Surface and TiO_2_

**Figure 3:**
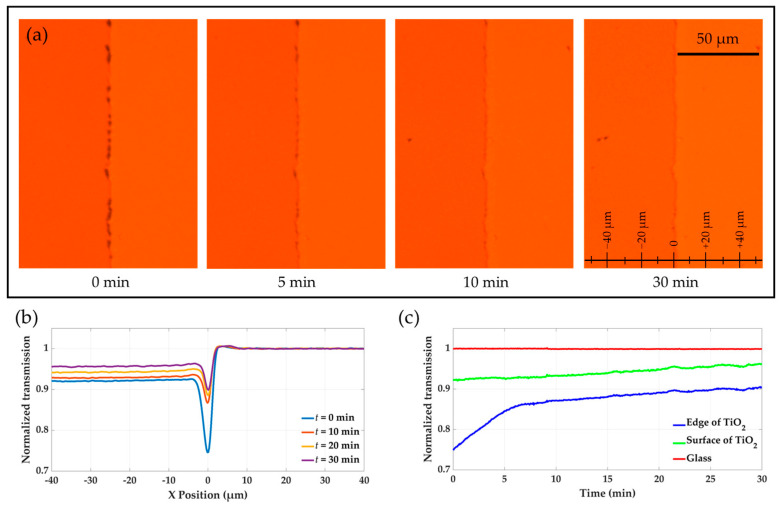
Real-time monitoring of gold dissolution (**a**) Sequential optical microscope images of a selected region around the TiO_2_ edge, including the TiO_2_ surface on the left side and the glass substrate on the right side, taken at various time points, *t* = 0, *t* = 5, *t* = 10, and *t* = 30 min, illustrating the gradual dissolution of gold structures over 30 min. (**b**) Transmission profiles across the TiO_2_ edge at *t* = 0, *t* = 10, *t* = 20, and *t* = 30 min, normalized to the reference transmission on the glass substrate. (**c**) Time-dependent transmission curves showing dissolution progress at the TiO_2_ edge and surface compared to the glass substrate.

Following the growth process, a KI solution diluted in DI water at a ratio of 1:300 was utilized to initiate chemical dissolution of the gold structures. The dissolution process was monitored using the same method as described for the growth process. Figure 3a displays the optical microscope images of a certain area around the TiO_2_ edge taken at different times during the dissolution process: *t* = 0, *t* = 5, *t* = 10, and *t* = 30 min. For a better visualization of the dissolution dynamics, a supplementary video (Video S2) is provided. Similar to the growth analysis, the TiO_2_ edge is located in the center, with the TiO_2_ surface and the glass substrate on the left and right sides, respectively. Figure 3b presents the transmission curves across the *x*-span of the selected region at four different time points: *t* = 0, *t* = 10, *t* = 20, and *t* = 30 min. All transmission values were normalized relative to the reference transmission on the bare glass substrate, located far from the TiO_2_ edge at *x* = 40 µm.

At the start of the dissolution experiment (*t* = 0), the transmission at the TiO_2_ edge (*x* = 0) was nearly 0.25 lower than that of the adjacent glass substrate (*x* > 0). This difference indicates that gold was substantially deposited along the edge of the TiO_2_. On the surface of the TiO_2_ (*x* < 0), the normalized transmission value was approximately 0.08 lower compared to the glass substrate, suggesting partial gold growth on the TiO_2_ surface. As dissolution proceeded, the gold structures dissolved at varying rates on the edge and surface of TiO_2_. While the transmission over the glass substrate remained constant throughout the experiment, a significant increase in transmission was observed on the TiO_2_ edge and surface as the gold structures dissolved.

The dissolution process was tracked on Figure 3c by monitoring transmission changes over time, with the glass substrate as a constant reference. Figure 3c shows time-dependent transmission curves, with a sharper increase at the TiO_2_ edge, indicating higher initial gold density, similar to the previous analysis.

### 2.3. Morphological Examination Using Scanning Electron Microscopy

In this section, the morphology of gold particles on a single sample was examined at different stages using SEM. The SEM imaging was performed using a Carl Zeiss Supra 55VP instrument (ZEISS AG, Oberkochen, Germany), operated at an acceleration voltage of 3 kV and a working distance of 3 mm. This technique provides high-resolution images that allow for a detailed visualization of the particles, revealing their size, shape, and distribution along the TiO_2_ patterns. By applying SEM, the structural characteristics of the gold particles could be analyzed, both after growth and after dissolution.

SEM analysis was first conducted to observe the morphology of the gold particles formed along the edges of the titanium dioxide patterns after 30 min of UV illumination. The photocatalytic growth experiment used a precursor solution of gold chloride mixed with isopropanol in a 10:1 ratio to enhance growth. After the growth phase, SEM was also performed following the chemical dissolution of the gold particles, where the KI solution was diluted with 300 mL of deionized water to assess the effects of dissolution.

#### 2.3.1. SEM Analysis of Grown Gold Particles on TiO_2_ Edges

**Figure 4:**
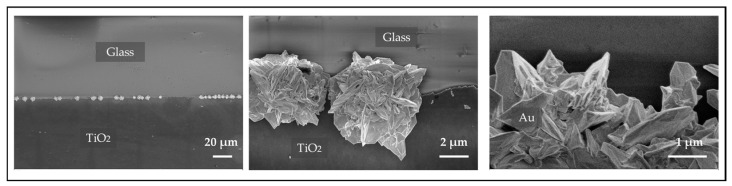
SEM images of the template post-growth, illustrating the formation of gold particles along the edges of the TiO_2_ patterns.

The SEM images in Figure 4 provide detailed insights into the selective growth of gold microstructures on the edge of TiO_2_ patterns. Notably, no visible particle growth is observed on the glass substrate, confirming that the photocatalytic deposition occurred exclusively on the TiO_2_. This result is significant, as the addition of isopropanol to the precursor solution, combined with the controlled intensity of the UV LEDs, prevented any unwanted nucleation or deposition on the glass, ensuring that growth was confined to the TiO_2_ regions, which is consistent with the in-situ microscopy measurements where the transmission intensity on the glass substrate remained unchanged during the growth process.

The gold microstructures display a distinct 3D flower-like morphology, primarily concentrated along the edges of the TiO_2_ patterns. These structures are composed of sharp, crossed plates, forming intricate flower-shaped formations. While the growth is focused along the edges, the microstructures do not form a continuous line; instead, they appear as separate clusters. This is due to the limited illumination time of 30 min in this experiment, as the focus was on studying the spontaneous growth and dissolution processes rather than forming a continuous line. With extended illumination times, a uniform and continuous gold line along the edges can be achieved [33]. In certain areas, however, the flower-shaped particles grow close together, nearly forming a connected structure along the edge. This unique morphology and spatial distribution suggest that the edges of the ITO-TiO_2_ patterns serve as preferential nucleation sites, promoting the formation of well-defined, three-dimensional gold structures.

#### 2.3.2. SEM Analysis After the Chemical Dissolution of Gold Structures

**Figure 5:**
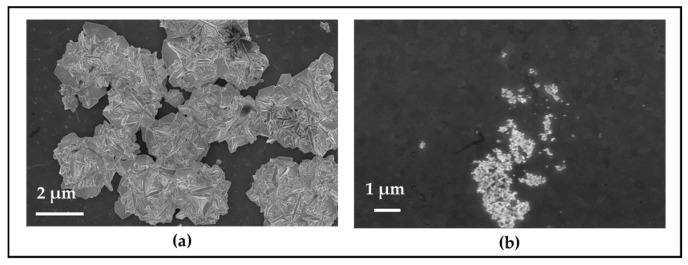
SEM images of gold particles on TiO_2_: (**a**) Before dissolution, with flower-shaped particles on the surface; (**b**) After dissolution, showing reduced and irregularly shaped particles, though not fully removed.

The SEM images in Figure 5, illustrate the morphological changes in the gold structures after the chemical dissolution process for 30 min. In some regions on the TiO_2_ surface, clusters of flower-shaped gold particles were observed to have grown, though these areas were limited both in number and in size. Figure 5a shows one of these small regions on the surface where the characteristic flower-shaped gold particles had formed prior to the dissolution experiment.

Following chemical dissolution using the KI solution, SEM imaging of the same area in Figure 5b reveals noticeable changes. The previously well-formed, flower-like gold particles appear etched and reduced in size, suggesting the dissolution process was partially successful. The gold structures seem to have been broken down into smaller fragments, though complete dissolution was not achieved, as some remnants of gold particles are still visible. The decrease in particle size and the etched morphology indicate that the KI solution effectively initiated the dissolution, but did not fully dissolve the gold structures.

This partial etching highlights the ability of the KI solution to interact with and reduce the size of gold particles, although further refinement of the dissolution conditions may be required to achieve full removal. Additionally, the size of the gold particles noticeably decreased, and their morphology transformed from the original flower-like shapes into more irregular, amorphous spots, indicating significant structural alteration during the dissolution process.

## 3. Discussion

Reconfigurable long-range connections with stimulus-induced formation and stimulus-free, spontaneous dissolution over time are in high demand to implement axon-like dynamic connection schemes. Possible technical implementations range from guided wiring upon metal filament formation in liquid media upon an electrochemical redox reaction [11] toward the electrophoretic reorganization of metallic nanoparticles into anisotropic long-range nanoparticle agglomerates along electrical field gradients in nanofluids [35]. In contrast to these earlier studies, which reported on material systems for the electrically stimulated growth of long-range connections, in this study, UV illumination is applied as a stimulus. To mimic a biological neural network, growth and dissolution rates should be compatible with each other. Photocatalytic gold growth allows for UV-stimulus activated connection formation. The rate may be tailored by adjusting the chemical composition of the precursor solution or by changing the UV wavelength or intensity. We enhanced the growth rate in this experiment by using isopropanol as a hole scavenger in the precursor solution. Additionally, the implementation of two UV LEDs, positioned at an angle for even illumination, further accelerated the growth process. We decreased the gold dissolution rate by diluting the KI solution. This had the additional benefit that a higher transparency is achieved, allowing for the real-time monitoring of the dissolution with the optical transmission microscope. Based on the results of a dilution series, the 1:300 dilution was chosen. The dissolution process in this setup was slightly slower than the gold growth. This is desired as the gold lines should grow under UV stimulus and dissolve without stimulus.

The incomplete dissolution of gold structures observed in this study is attributed to the slower dissolution rate relative to the growth rate under the chosen experimental conditions. While this resulted in residual gold particles, the dissolution process can be fully completed by increasing the concentration of KI in the DI water mixture. This adjustment would accelerate the dissolution, ensuring the complete removal of gold when required. However, in the context of mimicking axon-like behaviors in neuromorphic systems, achieving a fully resolved state may not be desired. Instead, the most interesting operation point is at the percolation threshold of the gold lines. By analyzing changes in conductance during the growth and dissolution processes, the system can effectively replicate the dynamic behavior of neural connections. This conductance-based approach provides insights into dynamic behaviors without necessitating complete dissolution, highlighting the versatility of the proposed system for neuromorphic and adaptive material applications.

The SEM observations confirmed gold growth and incomplete dissolution for 30 min of each process. Small amounts of gold residue remaining after the dissolution experiment confirm the lower dissolution rate compared to the growth rate. The time scales on the minutes to hours range align well with the remodeling times of biological systems. Further tuning of the rates is possible. Regarding long-range metal connection formation and dissolution, it has to be considered that a longer gold growth is necessary to achieve a conductive gold line without gaps. On the other hand, as soon as gaps appear, the electrical conductivity is lost. In future experiments, instead of pre-grown metal lines, a photo-forming step could be utilized to initiate long-range gold connections, similar to the electroforming process in filamentary memristive devices. Once a proto-filament is formed through the photo-forming step, subsequent cycles of UV illumination and dissolution can lead to dynamic reconfigurable states, mimicking axon-like connections. This approach aligns with the foundational role of forming steps in early memristive devices, as discussed in the reviews [36].

The diameter of the gold lines in our work is comparable to the diameter of biological axons in the micrometer range [37]. In biology, the plasticity of white matter is of high importance for learning and memory [38]. The conduction time of axons is not simply regulated by the axon diameter, but myelin formation and remodeling play a key role [39]. The inhomogeneous conductivity of biological axons may be compared to the inhomogeneous nature of our grown gold lines. The dissolution mimics the reversible and localized nature of axon pruning, focusing on the functional principles of dynamic connectivity rather than the molecular complexity of biological systems. Different to biology, our two-dimensional implementation has a much lower line density and total length. In a human brain, the combined length of myelinated axons reaches approximately 160,000 km [38]. Biological network reconfiguration times are on the time scale of hours to days and longer [38]. In our study, we considered growth or pruning sequences of half-hour durations for partial growth and partial dissolution. This is comparable to biological time frames. In summary, our proposed two-dimensional approach offers a similar line diameter as well as similar reconfiguration time scales, but a much shorter overall length. While acknowledging the significant differences, stimulus-adaptive gold “axon” formation and pruning are much closer to the biological situation than the fixed conductivity of electrical connections in standard electronics.

As in biology, this neuromorphic building block only functions in a liquid environment. This is highly unusual for electronic systems and definitely poses challenges regarding system stability, leakage, etc. Nevertheless, it is an intriguing thought to mimic axonal long-range connection growth and pruning, and the system opens the possibility to study the interaction of local UV stimuli and a homeostatic environment. Different than in biology, the liquid has to be exchanged between growth and dissolution. This poses an additional challenge for further development toward applications. We envision a microfluidic realization, where the liquid is exchanged in short intervals in a flow cell. Thus, this system is in principle suitable as a building block for neuromorphic engineering, allowing for sequential growth and dissolution cycles.

The findings of this study demonstrate a controlled approach to the photocatalytic growth and chemical dissolution of gold structures on a substrate, opening potential applications in neuromorphic systems. This study represents an effort to mimic axonal dynamics on a larger scale, providing a foundation for future work aimed at achieving finer and more biologically comparable structures [7]. The ability to dynamically grow and dissolve axon-like connections reflects fundamental biological processes, such as axonal growth and pruning, which are essential for synaptic plasticity and learning [10]. This capability could eventually lead to adaptive hardware systems that reconfigure in response to stimuli, offering a pathway toward more flexible and biologically inspired artificial neural networks.

Beyond neuromorphic systems, this method holds promise for bioelectronics, particularly for the development of reconfigurable sensor arrays. Dynamically adjustable conductive pathways could enable sensors to adapt in real time to changing environmental or physiological conditions [11]. Additionally, this approach offers a platform for studying synaptic behaviors in artificial systems, enabling controlled investigations of neural processes at a larger, more accessible scale.

While there are limitations in terms of achieving the high density of three-dimensional biological systems, the versatility of the proposed system lays a foundation for adaptive and responsive material systems. By bridging biological functionality and synthetic implementation, this work represents an important step toward biologically inspired material systems that operate on scales compatible with current fabrication technologies.

## 4. Materials and Methods

### 4.1. Substrate Prepration

In this study, a single type of substrate template was used for all experiments to investigate the photocatalytic growth and dissolution processes. Soda-lime glass substrates were precisely diced into 10 mm × 10 mm squares using a Wafer Dicing System (model DAD3350, Aurotech, Santa Rosa, Philippines) to ensure uniformity and provide a consistent surface area for material deposition. The substrates were then cleaned thoroughly to remove contaminants that could interfere with the photocatalytic reactions. The cleaning process involved sequential sonication in acetone and isopropanol (both of 99% purity, Sigma-Aldrich, St. Louis, MO, USA) in an ultrasonic bath (Martin Walter Ultraschalltechnik AG, Straubenhardt, Germany), followed by complete drying with high-purity nitrogen gas to ensure the removal of all solvent residues.

A 6 nm layer of ITO was deposited onto the cleaned glass substrates using physical vapor deposition (PVD) with an In_2_O_3_/SnO_2_ (90/10 wt%) target (99.99% purity, Kurt J. Lesker Company GmbH, Dresden, Germany). The ITO layer, selected for its higher work function relative to TiO_2_, was critical for achieving photocatalytic growth along the TiO_2_ edges [33]. The deposition of this thin, uniform, ITO layer formed a heterojunction TiO_2_-ITO interface, facilitating electron transfer and optimizing the conditions for gold growth during subsequent experiments.

Following ITO deposition, a 70 nm-thick layer of TiO_2_ was sputtered onto the ITO-coated substrates. This was achieved using a 3-inch TiO_2_ target (99.99% purity, Kurt J. Lesker Company GmbH) to ensure high-quality, uniform TiO_2_ coverage across the entire substrate. The TiO_2_ was deposited in its amorphous form, requiring further processing to transform it into its active photocatalytic anatase phase.

To pattern the TiO_2_ layer, a standard photolithography technique was employed. The substrates were first spin-coated with AZ5214E photoresist (Microchemicals GmbH, Ulm, Germany) at 3000 rpm for 30 s to achieve a uniform resist layer. Hexamethyldisiloxane (HMDS) was used as an adhesion promoter before applying the photoresist to ensure reliable patterning. After spin coating, the substrates were prebaked at 110 °C for 50 s to solidify the resist layer.

UV exposure was performed using a mask aligner (SUSS MicroTec, Garching, Germany), with a 5-inch photomask containing reflective chromium structures for line patterns as small as 50 µm (Rose Fotomasken, Bergisch Gladbach, Germany). The exposure energy was set to 32 mJ/cm^2^. Following exposure, a reversal bake at 120 °C for 2 min was performed to create the desired resist pattern, and a flood UV exposure with 320 mJ/cm^2^ was applied. The unexposed areas were developed using the AZ726 developer (Microchemicals GmbH) for 1 min, after which the substrates were rinsed with deionized water and dried with nitrogen gas.

The lift-off process, used to remove the unwanted TiO_2_ and ITO, involved immersing the substrates in acetone with ultrasonic agitation for 10 min, followed by a 5-min rinse in isopropanol. This process ensured that only the desired patterned regions of TiO_2_ remained on the ITO layer.

**Figure 6:**
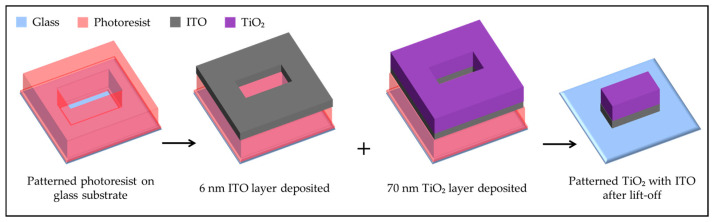
Schematic representation of the substrate preparation process. The glass substrate is negatively patterned with AZ5214E photoresist after lithography. A 6 nm ITO layer and a 70 nm TiO_2_ layer are then deposited via sputtering. After the lift-off process, the final patterned TiO_2_ structures with the underlying ITO layer are revealed.

Finally, to convert the sputtered TiO_2_ from its amorphous state to the photocatalytically active anatase phase, the substrates were annealed in a muffle furnace at 400 °C for 90 min. This heat treatment was critical for achieving the desired crystalline structure and ensuring optimal photocatalytic properties for the TiO_2_. After annealing, the substrates were rapidly cooled on a metal plate to lock in the anatase phase, preparing them for subsequent photocatalytic growth and dissolution experiments. The preparation process of the substrates, including lithographic patterning and material deposition, is schematically illustrated in Figure 6 to provide a clear overview of the procedural steps.

### 4.2. Photocatalytic Growth Experiment

After completing the heat treatment to convert the TiO_2_ to its anatase phase, the photocatalytic growth of gold lines along the edges of the TiO_2_ patterns was performed in a beaker. The precursor solution was prepared by dissolving 99.99% pure gold (III) chloride (HAuCl_4_) powder (Sigma-Aldrich) in deionized water, with a concentration of 15 mg in 60 mL of water. This solution was thoroughly mixed to ensure the complete dissolution of the gold chloride and to create a homogeneous precursor solution, essential for consistent photocatalytic growth across the entire surface of the substrates.

To enhance the growth rate of the gold particles and improve the efficiency of the photocatalytic process, the precursor solution was mixed with isopropanol in a 10:1 ratio. The addition of isopropanol, which acts as a hole scavenger, plays a critical role by accelerating the reduction of Au^3+^ ions under UV illumination.

**Figure 7:**
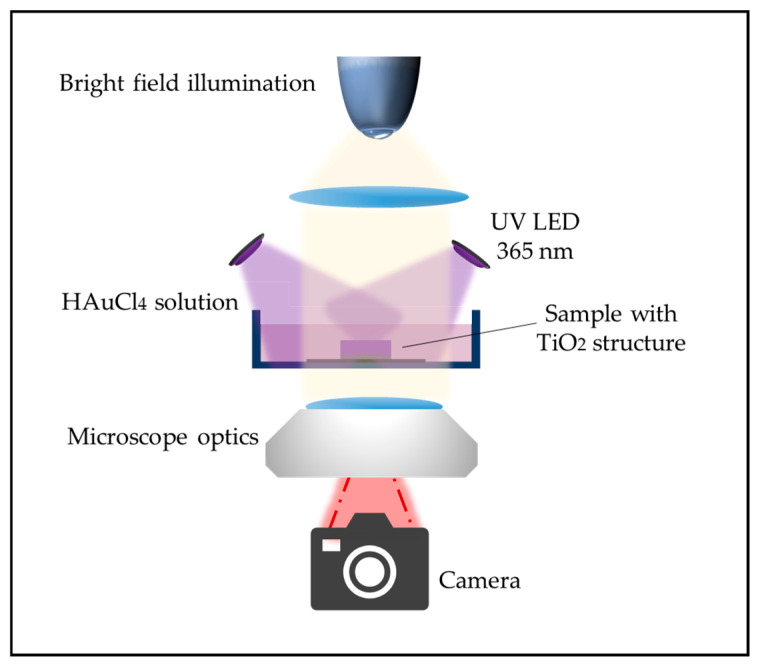
Schematic of the photocatalytic illumination setup. The sample with TiO_2_ structures is submerged in the HAuCl_4_ solution and illuminated by two angled UV LEDs (365 nm) under a transmission microscope connected to a camera.

Each substrate was positioned at the bottom of a glass beaker with the TiO_2_ facing upward. A total of 20 mL of the prepared precursor solution was added to the beaker, completely submerging the substrate. Two UV LEDs (Nichia, Tokushima, Japan), each emitting light at a wavelength of 365 nm, were positioned at opposite sides of the beaker with roughly 7 cm distance to the beaker, illuminating the template at an angle. The intensity of the UV light was measured using a Newport optical power meter and was found to be approximately 5 mJ/cm^2^.The total illumination time was set to 30 min, ensuring sufficient energy input for the reduction of Au^3+^ ions and the subsequent formation of gold lines along the edges of the TiO_2_ patterns. The schematic of the illumination experiment setup is shown in Figure 7. The setup is placed in a transmission microscope (DMi8 inverted microscope, Leica, Wetzlar, Germany) to allow for optical image recording of the gold growth and dissolution on the transparent substrates.

### 4.3. Dissolution Experiment Using a KI Solution Mixed in Different Ratios with DI Water

To investigate the chemical dissolution of gold lines grown on the edge of TiO_2_ patterns, a KI solution was prepared by mixing KI (Sigma-Aldrich, product number 204102) and iodine (I_2_) (Sigma-Aldrich) in a ratio of 1:4:40 (DI water), which is a commonly used ratio for gold dissolution in similar chemical systems [40]. After thoroughly mixing the KI and I_2_ with deionized water, the prepared solution was further diluted to different concentrations for the dissolution experiments.

**Figure 8:**
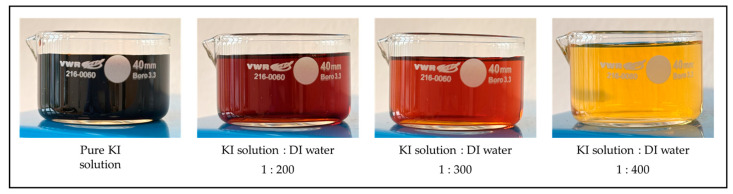
Visual representation of the KI solution diluted with DI water at different ratios. From left to right: pure KI solution, 1:200 KI solution to DI water, 1:300 KI solution to DI water, and 1:400 KI solution to DI water.

Three distinct dilutions of KI solution were prepared by mixing 1 mL of the stock solution with 200 mL, 300 mL, and 400 mL of DI water. These varying concentrations were chosen to explore how different KI solution strengths impact the dissolution rate of the gold lines. The prepared solutions, exhibiting different shades of color due to varying concentrations of KI in DI, are shown in Figure 8. As the KI solution is diluted with DI water, the color transitions from dark brown to a lighter reddish hue, becoming progressively lighter with increasing amounts of DI water.

After preparing the KI solutions, two substrates were used for the dissolution experiments. Both substrates first underwent the photocatalytic gold growth process as described in Section 4.2. For the dissolution experiment, one of the gold-grown substrates was immersed in the KI solution diluted with 300 mL of DI water, and the other substrate was immersed in the solution mixed with 400 mL of DI water. Both samples were kept in the solution for 30 min to allow sufficient dissolution of the gold lines. The dissolution experiments were conducted under a transmission microscope connected to a camera, enabling the real-time visualization and recording of the process.

### 4.4. Photocatalytic Growth and Chemical Dissolution Sequence

The experiment was conducted on the substrate described in Section 4.1, using a transmission microscope for the continuous real-time observation of both the photocatalytic growth and subsequent chemical dissolution processes. The substrate was fixed in place throughout the experiment, eliminating the need for repositioning during the solution exchange steps.

In the first step, the beaker was filled with 20 mL of a HAuCl_4_ precursor solution, which had been mixed with isopropanol in a 10:1 ratio to enhance the growth rate of the gold lines. The substrate, submerged in this solution, was then illuminated using two UV LEDs (365 nm), placed at an angle, for 30 min. This illumination initiated the photocatalytic growth of gold lines along the edges of the TiO_2_ patterns on the substrate. After the growth phase, the beaker and substrate were thoroughly rinsed with deionized water to remove any residual gold precursor solution, and the substrate was dried using nitrogen gas to prepare for the next step.

In the second step, the photocatalytically grown gold Structures were subjected to chemical dissolution. The beaker was filled with 20 mL of KI solution, diluted in 30 mL of DI water. During this phase, the UV LEDs were turned off to avoid any photocatalytic effects that could interfere with the dissolution process. The substrate remained in the KI solution for 30 min, allowing the KI solution to dissolve the gold lines by forming a soluble gold–iodide complex [40]. Following the dissolution, the substrate was again rinsed with deionized water to ensure the complete removal of the KI solution, and the substrate was dried using nitrogen gas.

## 5. Conclusions

In this study, we demonstrated the sequential photocatalytic growth and chemical dissolution of gold structures on a patterned ITO/TiO_2_ template. By optimizing the precursor solution, we achieved controlled gold growth along the edges of the ITO/TiO_2_ patterns and subsequently carried out the dissolution process in an optimized KI solution. The kinetics of the growth and dissolution processes were explored through real-time monitoring using optical transmission microscopy and image processing. Although a uniform gold line was not formed within the 30 min illumination, the focus of this study was on the sequential growth and dissolution processes rather than achieving continuous lines. While the current setup did not allow simultaneous growth and dissolution, the findings suggest potential for future microfluidic systems that enable dynamic solution exchange for UV-driven growth and dissolution cycles, mimicking biological processes such as axonal growth and pruning in bio-inspired networks. This work provides a robust methodology for optimized photocatalytic growth and dissolution processes, contributing to the development of dynamic, reconfigurable material systems for neuromorphic engineering and adaptive technologies.

## Data Availability

The data supporting the conclusions of this article will be made available by the corresponding authors on request.

## Supplementary Materials

The following supporting information can be downloaded at: https://www.mdpi.com/article/10.3390/molecules30010099/s1, Video S1: Gold growth dynamics in 30 min; Video S2: Gold dissolution dynamics in 30 min.
